# Complications associated with subsequent tunneled central venous access device placement in children: a retrospective cohort study

**DOI:** 10.1007/s00431-025-05985-1

**Published:** 2025-02-05

**Authors:** Ines Moraleda Guyol, Thanusiah Selvamoorthy, Ramsi Siaj, Julian Kolorz, Jan Sabo, Michael Berger, Julia Jeske

**Affiliations:** 1 Department of Pediatric Surgery, University Hospital Essen, University Duisburg-Essen, Essen, Germany; 2 Department of Pediatric Surgery, Children’s Hospital at Westmead, Sydney, Australia

**Keywords:** Central venous access device (CVAD), Central venous access surgery, Complications, Pediatrics, Pediatric surgery

## Abstract

Central venous access devices (CVADs) are vital instruments in pediatric healthcare, enabling the administration of critical treatments such as chemotherapy and parenteral nutrition. However, despite their advantages, CVADs carry a significant risk of complications, including infections, mechanical failures, and thrombotic events. From the current literature, it is unknown whether previous CVAD placements lead to an increased risk for complications in subsequent CVAD placements. We retrospectively analyzed data regarding tunneled, surgically implanted CVADs in children over a period of 2 years at a single tertiary pediatric center regarding their complications. Between 2021 and 2022, 328 CVAD implantations were performed in 313 children. The average age at implantation was 6.6 ± 5.5 years, while most of the patients were younger than 5 years old. During the study period, a total of 102 complications occurred in 96 patients. Most frequent complications were infections (18.29% of all implantations), followed by dislocation of the catheter tip (6.4 0%) and mechanical dysfunctions (4.27%). No patient died from a complication. The choice of catheter type (Port versus Broviac) showed a significant influence on the occurrence of complications (hazard ratio of 3.964 (95% CI 1.993–7.886; *p* < 0.001). The risk of infection and dislodgement was also higher when comparing Broviac with Ports implantations (infection: HR = 3.236; 95% CI 1.239–8.454; *p* = 0.017; dislodgement: HR = 5.781; 95% CI 1.229–27.193; *p* = 0.026). Cox regression showed a statistically significant higher risk of complications (especially infections) when the catheter was inserted via venous cutdown instead of percutaneous puncture technique (complications: HR = 6.709; 95% CI 1.776–25.337; *p* = 0.005; infections: HR = 7.28; 95% CI 1.096–48.379; *p* = 0.04). Cox regression did not show a statistically significant influence on complications for neither of the following factors: age, gender, weight, and oncological/non-oncological diagnosis. The complication rate for patients with previous CVAD was nearly the same as for patients who received a CVAD for the first time (previous CVAD: 29.17%; no previous CVAD: 30.74%).

*Conclusion*: Our study challenges conventional assumptions regarding the impact of previous CVAD placements on complication rates. Nevertheless, ongoing vigilance and adherence to standardized protocols remain crucial in mitigating risks and improving outcomes in pediatric CVAD management.

**Table:** 

**What is Known:** *• Risk factors predisposing for CVAD-related complications remain insufficiently understood.* *• It is unknown whether previous CVAD placements lead to an increased risk for complications in subsequent CVAD placements.* **What is New:** *• The complication rate for patients with previous CVAD appears to be the same as for patients who receive a CVAD for the first time.*

**What is Known:**

*• Risk factors predisposing for CVAD-related complications remain insufficiently understood.*

*• It is unknown whether previous CVAD placements lead to an increased risk for complications in subsequent CVAD placements.*

**What is New:**

*• The complication rate for patients with previous CVAD appears to be the same as for patients who receive a CVAD for the first time.*

## Introduction

Placement of a tunneled central venous access device (CVAD) is one of the most common procedures performed in pediatric surgery. CVADs are necessary for the appropriate treatment of a large variety of oncologic and non-oncologic chronic diseases because CVADs simplify regular blood sampling and permit repeated venous administration of medications, including chemotherapeutic agents. Other indications include parenteral nutrition, the chronic substitution of coagulation factors or blood products and enzyme therapy in metabolic diseases.

Despite their routine performance, CVADs have a high complication rate of roughly 25% [1, 2]. This high complication rate is multifactorial and is typically attributed to either catheter-related blood stream infection (BSI), dislodgement of the catheter tip, occlusion of the catheter, thrombosis formation around the catheter tip, arrhythmia due to irritation of the nerval structures of the heart, insertion site infection, and mechanical complication of the CVADs such as rupture as well as others. Even though CVADs have been placed in children at high frequencies for decades, risk factors predisposing for CVAD-related complications remain insufficiently understood. In recent years, both catheter type (Port versus Broviac), insertion site (femoral versus upper body), as well as final catheter tip position have been identified as risk factors for CVAD-related complications [1, 3, 4].

While general risk factors for CVAD complications are well studied, the cumulative impact of prior CVAD placements on subsequent complications remains unclear. Existing studies highlight challenges such as tissue scarring, reduced venous access, and infection risks; however, no study to date has systematically addressed the cumulative impact of prior CVAD placements on subsequent complications. Bridging this knowledge gap is essential, particularly in tertiary centers managing children with repeated CVAD needs.

We hypothesize that prior CVAD placement does not significantly increase the risk of complications, and we aim to answer the following research question: Does prior CVAD placement increase the risk of complications associated with subsequent CVAD implantation in children? Several studies explore general risk factors for CVAD complications, but there is a significant gap in the literature regarding the cumulative effects of prior CVADs on subsequent complications.

To our knowledge, no conclusive data exist regarding the question of whether previous CVAD placement imposes additional risk for children undergoing subsequent CVAD placement. Given our status as a tertiary referral center, our patient population has a high volume of multiple previous CVADs, rendering it an important potential risk factor. Therefore, in this study, we sought to investigate whether the complications rate of central venous access surgery is higher if children had previous CVADs.

## Materials and methods

### Patient cohort

Patients below 18 years of age undergoing CVAD implantation for various reasons were eligible for inclusion.

### CVAD systems and technique for vessel access

Single- or multiple lumen-tunneled central venous access devices (TCVADs), such as Broviac or Hickman catheters, Permcath, and completely implanted CVADs (Ports), were surgically implanted. Detailed analysis of CVADs included their material composition, presence or absence of antimicrobial coatings, and rationale for selection. In this study, only tunneled, surgically implanted CVADs such as Ports or Broviac catheters were included. Non-tunneled and peripherally inserted devices were excluded from the analysis. The procedures were performed under sterile conditions in aseptic operating rooms using mainly the following five venous access sites either through venous cutdown or percutaneous puncture following the Seldinger technique: internal jugular vein (via cutdown or puncture), external jugular vein (via cutdown), subclavian vein (via puncture), cephalic vein (via cutdown), or the anonymous vein (via puncture). Typically, a standard antibiotic, usually a first-generation cephalosporin, was administered within 30 minutes before the surgery.

### Catheter tip position

A chest X-ray was performed immediately following every implantation of a CVAD. The catheter tip position was defined as previously described [1]. Briefly, two conventional methods were used to categorize the location of the catheter tip, namely either a categorical division based on the position of the catheter tip relative to the right main bronchus or a metrical division based on the position of the catheter tip relative to the carina. Both methods are described elsewhere in detail [1].

### Data acquisition

This retrospective, single-center cohort study involved manual extraction of data from electronic medical records of pediatric patients with CVADs between 2021 and 2022. Demographic data, diagnosis specifics, implantation details (including CVAD type, caliber, and number of lumina), implantation techniques, venous sites, and catheter tip positions relative to anatomical landmarks were systematically collected.

The primary objective was to evaluate the impact of prior CVAD placements on complication rates. Other variables, such as catheter type, insertion technique, and patient demographics, were included as secondary analyses to control for potential confounders and provide a comprehensive risk assessment. Key data points included patient demographics (case number, birthdate, name, sex), clinical details (diagnosis date, weight at implantation), and procedural specifics (date of implantation, material composition). Additionally, prophylactic measures, complications (type, date), removal details (date, reason), and prior CVAD history were meticulously documented. Previous CVADs were defined as any tunneled CVAD implanted within the patient’s lifetime, with a recorded range of 1–5 prior placements.

Complications tracked encompassed bloodstream infections, site infections, dislodgements, occlusions, thromboses, arrhythmias, mechanical issues, and bleeding events.

### Definition of complications

Complications were categorized as follows: catheter-related bloodstream infections (BSI), catheter dislodgement, occlusion, thrombosis, arrhythmia, insertion site infections, mechanical issues, and others. Immediate minor issues such as brief local bleeding at the implantation site that had no subsequent consequences in medical care were not considered.

Catheter-related BSI was diagnosed based on clinical signs of systemic infection and at least one positive blood culture obtained via the CVAD. Removal of the contaminated catheter depended on factors such as infection severity, pathogen type, and response to antibiotic treatment and was always discussed together with the primary team (e.g., the pediatric oncologists). Dislodgement referred to any shift in the catheter tip position away from the central part of the venous system, rendering it unusable as a CVAD. CVAD occlusion was identified by partial or complete blockage of the catheter lumen, requiring surgical intervention for resolution. Thrombosis involved the formation of a clot extending to the nearby vessel area, including potential pulmonary embolism. Mechanical complications encompassed any dysfunction of the CVAD and included rupture or other damages to the CVAD.

In cases where two complications occurred at the same time, one complication was defined as the main complication leading to the other one (mechanical complication leading to dislodgement and thrombosis, thrombosis leading to occlusion).

### Statistics

Continuous variables were expressed as median or mean ± standard deviation and categorical variables as frequency (%). Cox proportional regression was used to investigate the relationship of complication rates and other parameters of interest. Patients who did not experience the outcome event were censored at the time of the last follow-up. We rigorously tested the assumptions of the Cox proportional hazards model, including checks for proportionality, to ensure the validity of our findings. Additionally, sensitivity analyses were conducted to assess the robustness of the results, particularly in subgroups with repeated CVAD placements. These measures strengthen the reliability of our conclusions despite the retrospective nature of the study. Hazard ratios were estimated with 95% CIs and a multivariable model was constructed. Results were considered statistically significant at *p* < 0.05. Data in this study was analyzed using IBM SPSS Statistics 29.0.02(20).

### Ethics statement

This study was conducted in accordance with the Declaration of Helsinki and approved by the Ethics Committee of the University Hospital Essen (Approval number: 22–10,967-BO). The Ethics Committee emphasized that, due to the absence of consent, only retrospective data from existing medical records obtained through routine clinical care may be used for this research. In compliance with data protection regulations, personal data will be anonymized as soon as the research purpose allows.

### Consent to participate

As this is a retrospective study, no informed consent was required.

## Results

### Biometric and clinical data

**Fig. 1:**
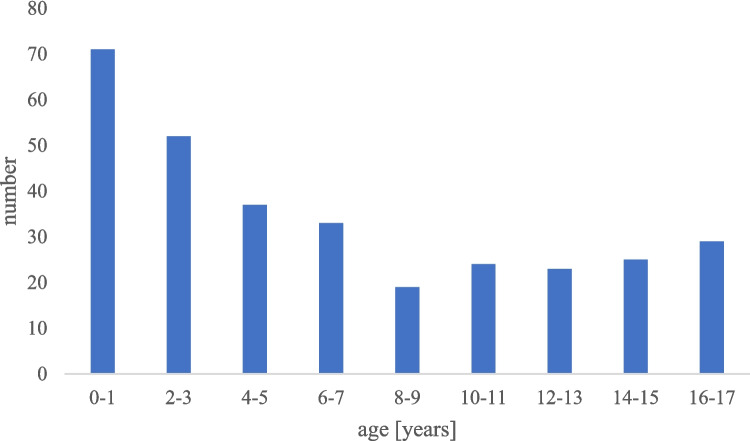
Age distribution of central venous catheters implanted. The average age at implantation was 6.6 ± 5.5 years with the youngest patient being 2 weeks old

The study covers catheter implantations that took place at our center in 2021 and 2022 and included 328 catheter implantations in 313 pediatric patients. The average age at implantation was 6.6 ± 5.5 years, while most of the patients were younger than 5 years old with the youngest patient being two weeks old (Fig. 1). Sixty percent of the patients were male. The underlying diseases can be divided in oncological (82.11%) and non-oncological cases (17.89%).

### Catheter type and site of implantation

In most children, a port was implanted (57.32% of cases), followed by the implantation of a Broviac catheter (36.59%) and a Permcath (6.1%). The most frequently used technique was venous cutdown (59.45% of all implantations) versus percutaneous puncture technique (31.70%). Approximately two-thirds of the implantations were done on the left side (66.16%). The most frequently used vein was the cephalic vein (36.28%), followed by the subclavian vein (31.40%) and the external jugular vein (24.70%). In the case of port implantation, the cephalic vein (61.70%) was most frequently chosen, while for Broviacs, subclavian vein (50.0%) and external jugular vein (38.33%) were most often selected.

At the end of this study, 163 catheters remained implanted by reason of ongoing therapy. The remaining catheters had been removed due to the following reasons: finished therapy (69 catheters; 21.04%), complications (91 catheters; 27.74%) or other reasons (five catheters; 1.52%).

### Catheter tip position

In more than half of the implantations, the catheter tip was located below the right main bronchus (50.91%, “deep”), while in one third of the cases (30.18%) the catheter tip was located at the same level of the right main stem bronchus (“middle”) and in roughly one fifth of the cases above it, (“high”, 18.29%). The average distance to the carina was 2.65 cm ± 1.52 cm.

### Complications

During the study, a total of 102 complications occurred in 96 patients. Of all 328 catheters, 91 catheters (27.74%) had to be removed due to a complication. Most frequent complications were infections (18.29% of all implantations), followed by dislocation of the catheter tip (6.40%) and mechanical dysfunctions (4.27%). Rare complications were thrombosis (0.91%) as well as occlusion and bleedings (0.61% each). No patient died from any of the described complications.

The median time until a complication occurred was 108.5 days. Of all complications, dislodgements occurred the earliest with a median of 31 days, while occlusions occurred latest in median after 173 days. Infections appeared in median after 131 days and mechanical dysfunctions after 96 days.

The choice of catheter type (Port versus Broviac) showed a significant influence on the occurrence of complications (Table 1). While in 59 of the 120 cases where a Broviac was implanted a complication occurred (49.17%). For Port implantations, complications were observed in only 36 of the 188 cases (19.15%). Cox regression analysis showed an increased risk of complications when a Broviac catheter was implanted versus that of a Port catheter with a hazard ratio of 3.964 (95% CI 1.993–7.886; *p* < 0.001).

**Table 1:** Multivariate analysis shows dependency of complications on catheter type

Complications	No. (%) Total (*n* = 328)	No. (%) Port (*n* = 188)	No. (%) Broviac (*n* = 1 20)	Hazard ratio (95% CI)	*P* value
Overall	102 (31.1)	36 (19.15)	59 (49.17)	3.964 (1.993–7.886)	< 0.001
Infection	60 (18.29)	20 (10.64)	36 (30.0)	3.236 (1.239–8.454)	0.017
Dislodgement	21 (6.4)	5 (2.66)	15 (12.5)	5.781 (1.229–27.193)	0.026

*CI* confidence interval

The risk of infection and dislodgement was also higher when comparing Broviac with Port implantations (infection: HR = 3.236; 95% CI 1.239–8.454; *p* = 0.017; dislodgement: HR = 5.781; 95% CI 1.229–27.193; *p* = 0.026; Table 1). Cox regression showed a statistically significant higher risk of complications (especially infections) when the catheter was inserted via venous cutdown instead of percutaneous puncture technique (complications: HR = 6.709; 95% CI 1.776–25.337; *p* = 0.005; infections: HR = 7.28; 95% CI 1.096–48.379; *p* = 0.04), although venous cutdown is more frequently used in combination with Ports (78.19% of all Port implantations; 39.17% of all Broviac implantations).

Moreover, using a CVAD with a higher caliber led to a slightly increased risk of getting an infection (HR = 1.287; 95% CI 1.038–1.596; *p* = 0.022).

Cox regression did not show a statistically significant influence on complications for neither of the following factors: age, gender, weight, and oncological/non-oncological diagnosis.

### Influence of previous catheterization on complication rates

**Fig. 2:**
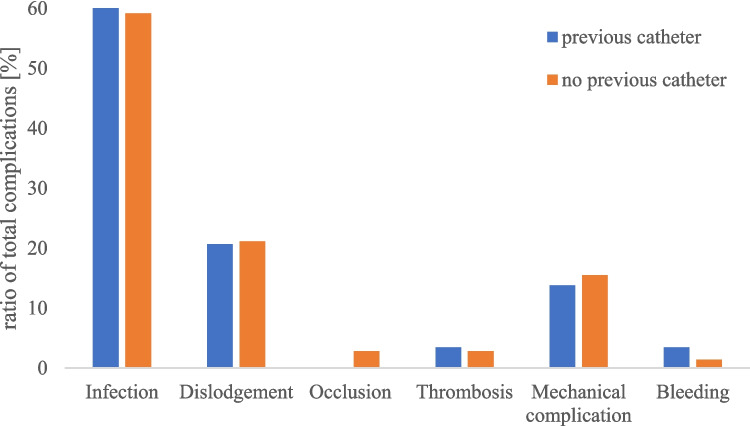
Complication rates in dependency of previous CVADs. The occurrence of the examined complications did not differ in patients with previous CVADs

In this study, 96 children had a previous CVAD, varying from one to five previous CVADs. The complication rate for patients with previous CVAD was nearly the same as for patients who received a CVAD for the first time (previous CVAD: 29.17%, 28 patients; no previous CVAD: 30.74%, 71 patients). There was also no difference in the type of complication: in both cases, infections were most frequent (> 50%), followed by dislodgement (previous CVAD: 20.69%, no previous CVAD: 21.13%) and mechanical complications (previous CVAD: 13.79%, no previous CVAD: 15.49%; Fig. 2).

## Discussion

Central venous access devices (CVADs) are indispensable tools in pediatric healthcare, facilitating essential therapies ranging from chemotherapy to parenteral nutrition. Despite their benefits, CVADs are associated with a considerable risk of complications, including infections, mechanical issues, and thrombotic events [1, 3, 4]. Understanding factors that influence these risks is critical for optimizing patient care. In this study, we retrospectively analyzed data regarding 328 CVAD implantations in children over a period of 2 years at a tertiary pediatric center regarding their complications. Given the lack of its information in the published literature, we specifically analyzed whether a previous CVAD implantation had a higher complication rate in children compared to children undergoing their first CVAD implantation. To our knowledge, this was the first time to address this question specifically.

Overall, our complication rate was similar to data published by others [1, 3, 4]. Hofmann et al. recently described and analyzed the complications of 396 CVAD implantations in children in a single institution over a 10-year period [1]. Although their slightly higher number of patients was collected over a much longer time period, their cohort and its data nevertheless allow for good comparison. In their cohort, similar to ours, infections (36.4%) were the most prevalent complication. Also similar to our data, catheter tip position or vessel insertion site had no significant impact on the risk of developing a complication. Likewise, compared to our data, in their cohort there was no death related to a CVAD implantation. Different from our study, they did not assess whether the implantation of a previous CVAD had an impact on the rate of complications.

Analogous to other studies, our study examined the distribution of complications, finding infections to be the most common issue in both groups (> 50%), followed by catheter dislodgement and mechanical complications. The observed complication rates in our cohort align with those reported in previous studies, which indicate that CVAD-associated infections remain the predominant complication in pediatric populations [1, 5, 6]. These findings underscore the importance of stringent infection control protocols regardless of the patient’s history with CVADs [7].

From the current literature, it is unclear whether repeat CVAD placements predispose patients to a higher risk of specific complications. Intuitively, one could easily make the argument that technical challenges related to repeat vascular access surgery, limited availability of access sites and both local and vascular scarring in children undergoing multiple CVAD placements inherently increase the complication rate. On the other hand, advancements in surgical techniques, such as ultrasound-guided insertions and the use of antimicrobial-impregnated catheters (which we did not use in this cohort), may mitigate these risks and contribute to improved outcomes. Moreover, the materials used in CVADs have evolved, with modern catheters exhibiting enhanced biocompatibility and reduced infection rates [8]. These advancements, coupled with rigorous infection control measures, likely play a significant role in the influence on complication rates observed between patients with and without prior CVAD placements.

Given that our center is a super-regional tertiary referral center with highly complex pediatric patients, a scenario of a pediatric patient needing multiple catheters is a rather common occurrence. In the cohort analyzed here over a period of only 2 years, 96 children (approximately one-third of children) had between one and five previous CVAD placements. Hence, when analyzing our cohort, we specifically focused on this question.

Interestingly, our findings revealed no significant difference in overall complication rates between patients with a history of previous CVADs and those undergoing their initial CVAD placement. Specifically, complication rates were comparable at 29.17% among patients with prior CVADs and 30.74% among those without previous placements. Therefore, our study challenges the conventional assumption that multiple CVAD placements inherently elevate the risk of complications. Consequently, regarding additional CVAD placements following previous CVADs, each child’s individual situation should continue to serve as the basis when assessing the risk profile of CVAD placement.

Our study has limitations. As a retrospective analysis from a single center, our findings may not fully capture the complexity and variability encountered across different healthcare settings and patient populations. Additionally, our study did not fully account for potential confounding factors such as variations in patient anatomy, underlying health conditions, and differences in post-implantation care. Also, as a retrospective study, this research is subject to the potential for selection bias and confounding factors such as disease severity and variations in post-implantation care. We addressed these biases by using multivariable regression models to adjust for known confounders. Additionally, we performed sensitivity analyses and tested for proportional hazard assumptions to strengthen the robustness of our findings. Nonetheless, prospective studies are warranted to validate these results further.

In conclusion, while our study challenges conventional assumptions about the impact of previous CVAD placements on complication rates, ongoing vigilance and adherence to standardized protocols remain crucial in mitigating risks and improving outcomes in pediatric CVAD management. Further research is necessary to confirm our findings and to continue advancing the care of pediatric patients requiring central venous access.

## Data Availability

No datasets were generated or analysed during the current study.
